# Impact of the presence of partially erupted third molars on the local radiographic bone condition

**DOI:** 10.1038/s41598-022-12729-w

**Published:** 2022-05-23

**Authors:** Ighor Andrade Fernandes, Endi Lanza Galvão, Patricia Furtado Gonçalves, Saulo Gabriel Moreira Falci

**Affiliations:** Section of Oral and Maxillofacial Surgery, Department of Dentistry, Universidade Federal dos Vales do Jequitinhonha e Mucuri, Rua da Glória, 187-Centro-Clínica de Cirurgia Bucal, Diamantina, Brazil

**Keywords:** Dental diseases, Periodontitis, Oral anatomy, Dental radiology, Periodontics

## Abstract

The decision on retaining or prophylactically removing asymptomatic lower third molars is still discussed in the literature. This study aimed to verify the association between asymptomatic lower third molars and local bone conditions through periapical radiographs. Based on sample size calculations, 288 radiographs were required. Dependent variables were alveolar bone crest status and radiolucency between the distal aspect of the third molar crown and the ascending mandibular ramus. Independent variables were sex, age, Pell and Gregory and Winter’s classification, angulation and distance between second and third molars, third molar side. Advanced ages (OR 1.15; CI 1.08–1.24; p < 0.001) and greater third molar angulations (OR 1.03; CI 1.01–1.04; p < 0.001) significantly increased the chance of radiographic alterations in the bone crest between second and third molars. Radiolucency distal to third molars was solely impacted by patient’s age (OR 1.05; CI 1.01–1.11; p = 0.036). Older patients and lower third molars with greater angulations about adjacent second molar should be evaluated for third molar removal because of the increased chance of alveolar bone crest alterations. Older patients should also be monitored for wider radiolucent pericoronal spaces distal to lower third molars and its consequences.

## Introduction

Many young adults have at least one-third molar (3M) and it is common the occurrence problems related to the presence of these teeth. Specifically, partially erupted lower 3Ms may predispose some complications to the adjacent dentition and structures, especially second molars (2M), such as distal cervical caries of the 2M^1–5^, periodontal pathologies^4^, and root resorption^5^.

Periodontal problems due to the presence of lower 3Ms are related to distal bone resorptions of the adjacent 2M and/or external root resorptions of 2M^4^, and a periodontal inflammatory disease affecting 3Ms^6^. Data derived from the Shanghai Xuhui District Dental Disease Prevention and Control Institute showed that mesioangular and horizontally impacted 3M in relative deep positions were associated with periodontal pathologies in the distal aspect of the 2M, while those soft tissue impacted and vertically angulated 3M were more associated with pericoronitis. However, the decision to remove or retain these teeth, when faced with asymptomatic patients with what appears to be a healthy periodontium upon visual inspection, is still a dilemma for dental surgeons.

The evaluation of periodontal bone structure through radiographic examinations becomes an efficient alternative approach for detecting changes caused by 3Ms once it is deemed a minimally invasive procedure^7^. Polat et al.^8^ reported the association between position and angulation of lower 3Ms and periodontal pathologies in adjacent 2Ms. Li et al.^9^ reported a greater chance (OR 1.35 to 2.44) of bone loss distal to 2M adjacent to partially erupted lower 3M when compared to 2Ms without adjacent 3M. However, these studies evaluated panoramic radiographs, which are efficient in planning dental surgeries^1^ but are less accurate for the assessment of alterations in periodontal bone structures^10^.

Although periodontal probing remains the gold standard for periodontal assessment, it has limitations regarding reproducibility and sensitivity^11,12^. On the other hand, radiographic examination and assessment of alveolar crest levels around individual teeth is a useful diagnostic adjunct to clinical periodontal examination^12^. It allows the accurate evaluation of crestal-bone architecture, crown-root ratios, presence of vertical or horizontal bone defects, furcation involvements, and the overall bone morphology^12^. To the authors’ knowledge, studies evaluating the presence of 3M and their influence on local bone architecture through periapical radiographs are not available. Thus, research with an appropriate methodology to test the hypothesis of this association is necessary.

The objective of this study was to establish whether there is an association between the presence of partially erupted 3Ms and bone conditions (alveolar bone crest and distal radiolucency) in the 3M region through periapical radiographs analysis. The null hypothesis of an absence of any relationship among local bone structures and the presence of partially erupted lower 3Ms was studied.

## Methods

This was a cross-sectional study approved by the Research Ethics Committee of the *Universidade Federal dos Vales do Jequitinhonha e Mucuri* (Brazil) (Reference number: 1.952.362) on March 7th, 2017 and was conducted following the Strengthening the Reporting of Observational Studies in Epidemiology (STROBE) statement. All the methods were performed in accordance with the relevant guidelines and regulations. All radiographs used in this study were obtained from archives of patients from the university clinics in which an informed consent form is signed by all the patients before any intervention, highlighting the possible use of materials from their records for research purposes. Sample size calculation was performed through the population proportion confidence interval formula. The prevalence rate of periodontal pocket distal to 2Ms adjacent to 3Ms was used^13^, assuming the value of 25% (0.25), admitting an error of 5% (0.05), and confidence level of 95%. Thus, 288 periapical radiographs were necessary for this study.

Patients’ records containing high-quality periapical radiographs exhibiting high contrast and satisfactory relationship among 2Ms and partially erupted lower 3Ms (including roots and crowns), and ascending mandibular ramus, were included in the sample. Radiographs with inadequate exposure and fixation were excluded, as were those collimated and with distortion. Radiographs of fully erupted or impacted lower 3Ms or showing overlapping enamel and restorations on the adjacent 2M that would make it difficult to analyze the variables were also considered inadequate for the study.

Radiograph selection was carried out from the analysis of dental records of patients who underwent surgical removal of lower 3Ms at the Oral Surgery Clinic of the referenced university. Clinical records were assessed until the total number of 288 radiographs was attained. To reach the sample, 1326 clinical records from 1999 to 2018, were analyzed. Since 288 radiographs that met the eligibility criteria became the required sample, randomization was not necessary.

Dependent variables were alveolar bone crest alterations between 3 and 2M and radiolucency between the distal aspect of the 3M crown and mandibular ramus (pericoronal/ follicular sac space region). Between the 3M and 2M, the bone crest status was categorized in the absence or presence of alterations. This is an important aspect to verify since it may be a sign of the initial process of periodontal diseases^11^. The alterations consisted in the presence of a blurry bone aspect, horizontal bone loss, or vertical bone loss. The radiolucency between the distal aspect of the 3M crown and mandibular ramus was evaluated according to the following categories: 0 to 1.99 mm and 2.00 to 4.10 mm. Although controversial in the literature, the 2 mm value was used as the cut-off point for analysis once a radiolucent area wider than 2 mm may be associated with a pathological process^14^.

**Figure 1:**
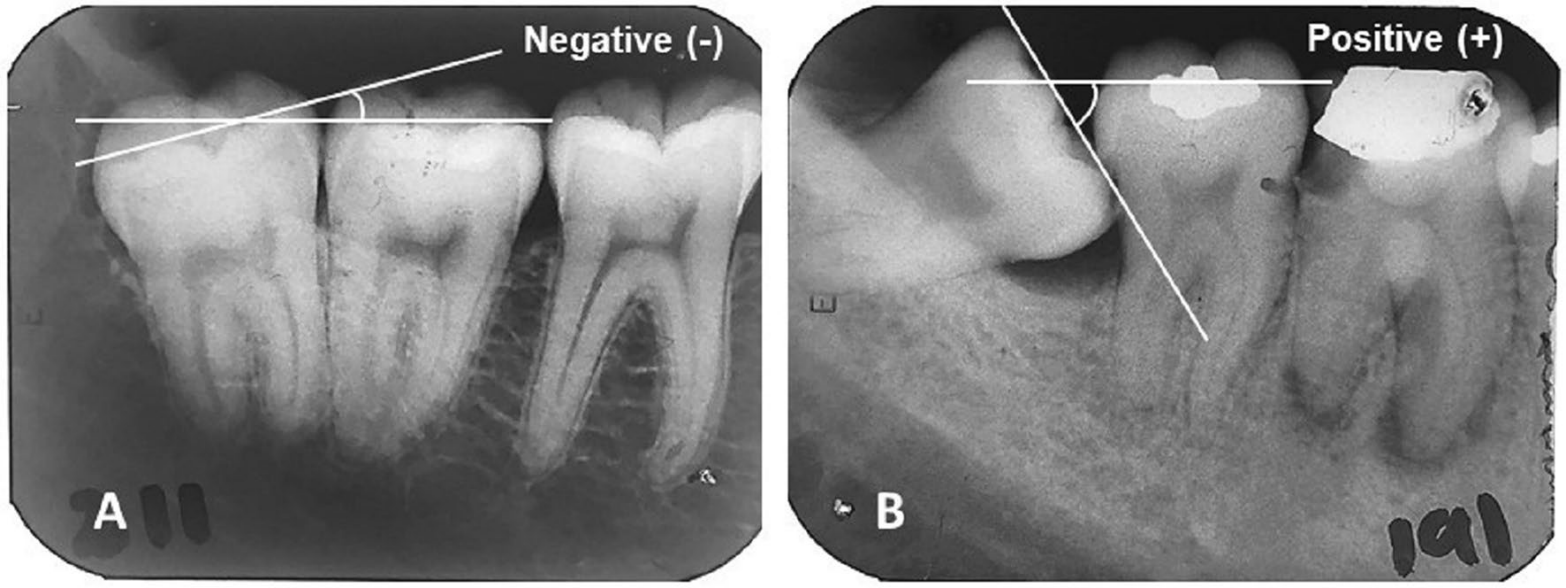
Angulation between lower 3 M and adjacent 2 M. A line drawn parallel to the 3 M occlusal plane, touching the tips of the cusps and traced along the mesiodistal axis of the tooth, was used as a reference. A similar line drawn parallel to the adjacent 2 M occlusal plane intersects the reference line and creates an angle. (**A**) The angle created below the reference line is classified as negative. (**B**) The angle created above the reference line is classified as positive.

**Figure 2:**
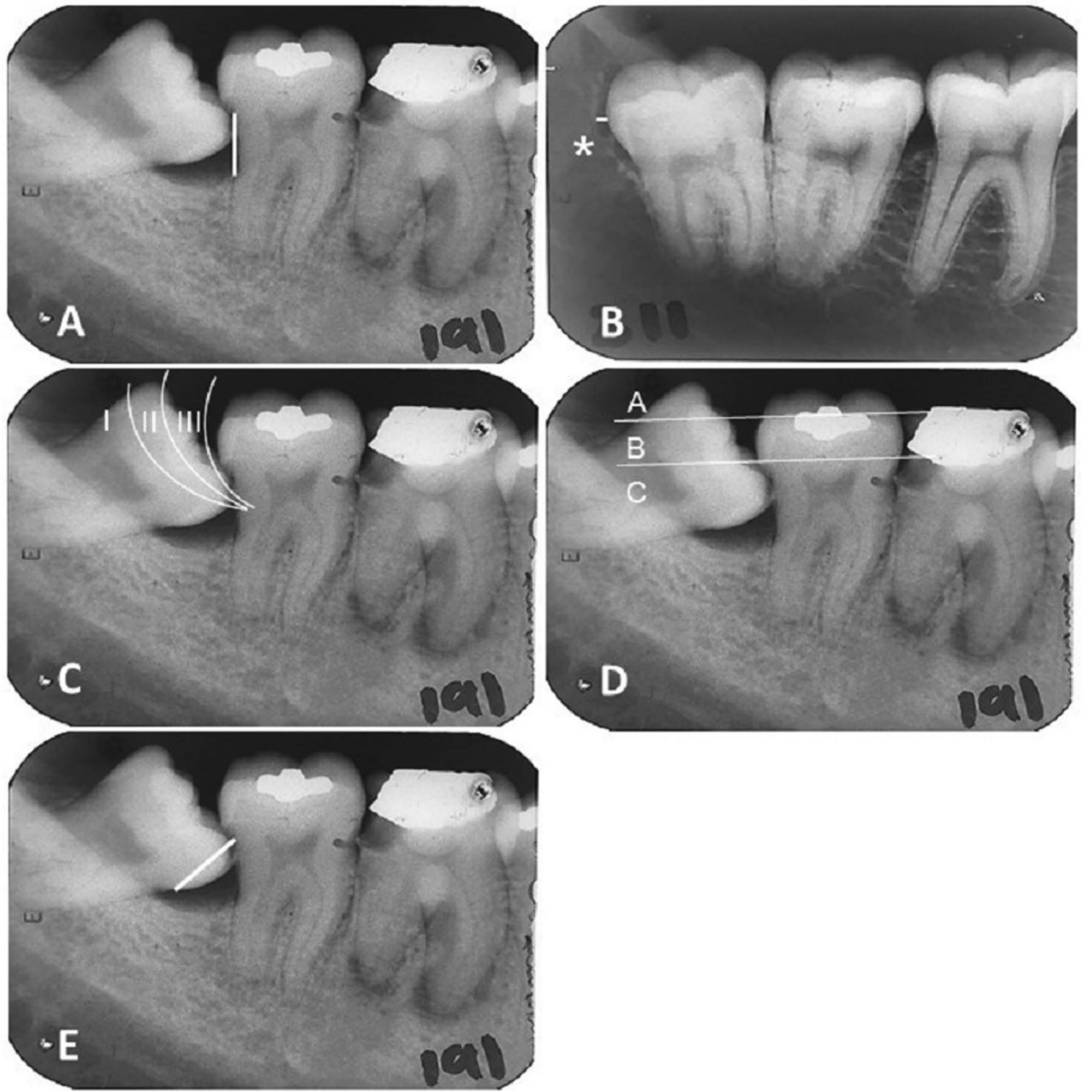
Variables’ descriptions. (**A**) Bone loss was evaluated according to the distance (mm) between CEJ and present marginal bone level in the distal aspect of lower 2Ms when in the presence of adjacent 3Ms. (**B**) *Radiolucency between distal aspect of lower 3M crown and mandibular ramus was measured as the distance (mm) between the extremity of the distal aspect of the dental crown and the more distant point of bone resorption. (**C**) Pell and Gregory classification about mandibular ramus: Class I—mandibular ramus located distal to the 3M; Class II—mandibular ramus located between the distal surface and middle of the lower 3M crown; Class III—mandibular ramus located between the mesial surface and middle of the lower 3M crown. (**D**) Pell and Gregory classification about the occlusal plane: Class A—3M occlusal plane is located at the same level or above the occlusal plane of the adjacent 2M; Class B—3M occlusal plane is located between occlusal plane and cervical line of the adjacent 2M; Class C—3M occlusal plane is located below the cervical line of the adjacent 2M. (**E**) Leone classification: distance (mm) between 2 and 3M is determined by the distance between CEJ of the mesial aspect of the 3M and CEJ of the distal aspect of the adjacent 2M.

The independent variables were: sex, age, angulation between 3 and 2M (Fig. 1), side of the 3M (right of left); distance between the cementoenamel junction (CEJ) and alveolar bone crest level distal to 2Ms (Fig. 2A), periodontal ligament width, radiolucency on the distal surface of 3 M (Fig. 2B); Pell and Gregory classifications (Fig. 2C,D), Winter’s classification, distance from the CEJ of the mesial aspect of the 3M to the CEJ of the distal aspect of the adjacent 2M (Fig. 2E). A previously calibrated examiner (ICC: 0.65–0.99; kappa: 0.72–0.99) was responsible for collecting the variables. The radiographs were evaluated using a negatoscope.

To measure angulation between 2 and 3M, the radiographs were scanned, and measurements were taken through two lines drawn in the PowerPoint (Microsoft Office 2016, Microsoft, Albuquerque, NM, US) (Fig. 1). A line drawn parallel to the 3M occlusal plane, touching the tips of the cusps and traced along the mesiodistal axis of the tooth, was used as a reference. A similar line drawn parallel to the adjacent 2M occlusal plane intersects the reference line and creates an angle. The angle formed at the intersection of the two lines was measured using a protractor (Faber-Castell, Stein, Germany). Negative angles were related to 3Ms tending to disto-angulation, and the positive ones were related to those tending to mesio-angulation. Dependent variables and the distance between CEJ’s of 2M and 3M were assessed with a digital caliper assisted by a magnifier. In the data collection process, at every 30 min of radiographic evaluation, the examiner took a break of 15 min to avoid visual disturbances.

To analyze the data, the program R (version 3.6.2, R Core Team, Vienna, Austria, www.r-project.org) was used, using the “caret” and “mtest” packages. Univariate logistic regression models were used to verify associations between independent and dependent variables, and the *p*-value was set at < 0.20 to include the variables in multivariate analyzes. The selection of the variables and the most appropriate multivariate model was conducted with both backward and forward stepwise analysis to further explore those variables significantly associated with alveolar bone crest status and radiolucency between the distal aspect of the lower 3M crown and mandibular ramus. Models were compared using the Akaike information criterion (AIC). The variables which were retained in the final step of the model were reported in terms of odds ratio (OR) and corresponding 95% confidence interval (CI).

### Ethical approval

This study was approved by the Research Ethics Committee of the Universidade Federal dos Vales do Jequitinhonha e Mucuri (Reference number: 1.952.362).

## Results

The mean (± SD) age of the patients was 23.10 years (± 5.55; range 15–57). Radiolucency on the distal aspect of the lower 3Ms was 0.87 mm (± 0.96; range 0–4.10). Periodontal ligament width distal to 2Ms was 0.08 mm (± 0.03; range 0–0.20). The average (± SD) angulation between 2 and 3M was 8.50 degrees (± 30.16; range − 27 to 99), and the mean (± SD) distance between them was 3.41 mm (± 2.36; range 0.51–12.09). The average (± SD) marginal bone level distal to 2Ms was 1.83 mm (± 1.81; range 0–10.44).

In the univariate logistic regression using alveolar bone crest alterations as the dependent variable, sex (p = 0.053), age (p < 0.001), angulation between 3 and 2M (p < 0.001), the distance between 2 and 3M (p < 0.001), and Pell and Gregory classification for mandibular ramus (p = 0.019) and occlusal plane (p = 0.012), were included in the multivariate analysis (Table 1). The multivariate logistic regression showed that advanced ages (OR 1.15; CI 1.08–1.24; p < 0.001) and greater angulations between the 3M and the adjacent 2M (OR 1.03; CI 1.01–1.04; p < 0.001) increase the chance of alveolar bone crest alterations when compared to younger ages and smaller angulations (Table 1).

**Table 1:** Frequency distribution plus univariate/multivariate logistic regression model: Bone crest status and independent variables.

	Total (n)	Alveolar bone crest status		Univariate OR (CI 95%)	*p*-value	Multivariate OR (CI 95%)	*p*-value
		Preserved	Altered				
**Sex**							
Male	102	84	18	1	0.053*	1	0.140
Female	186	168	18	0.50 (0.24–1.01)		0.52 (0.22–1.23)	
Age (years)	288	22.35 (± 4.36)	28.39 (± 9.13)	1.16 (1.09–1.24)	< 0.001**	1.15 (1.08–1.24)	< 0.001**
Angulation between 3 and 2M	288	16.34 (± 28.65)	48.33 (± 25.51)	1.03 (1.02–1.04)	< 0.001**	1.03 (1.01–1.04)	< 0.001**
Distance from CEJ (mm)	288	3.10 (± 2.25)	5.49 (± 2.02)	1.47 (1.27–1.71)	< 0.001**	–	–
**Third molar**							
38 (left)	138	119	19	1		–	–
48 (right)	150	133	17	0.80 (0.39–1.61)	0.533	–	–
**Pell and Gregory (occlusal surface)**							
I	121	113	8	1	0.012**	1	0.085
II	167	139	28	2.84 (1.30–9.91)		2.27 (0.92–6.14)	
III	0	0	0	–			
**Pell and Gregory (ramus)**							
A	48	37	11	1	0.019**	–	–
B	240	215	25	0.39 (0.18–0.88)		–	–
C	0	0	0	–		–	–
**Winter classification**							
Vertical	129	125	4	1		–	–
Mesioangular	93	71	22	1.21 (0.52–2.77)	0.640	–	–
Horizontal	28	19	9	1.78 (0.53–5.19)	0.308	–	–
Distoangular	38	37	1	1.24 (0.37–3.52)	0.695	–	–

Data are presented as n of subjects (%) or as mean ± SD.

*OR* odds ratio, *CI 95%* 95% of confidence interval.

*p < 0.20; **p < 0.05.

On the other hand, only age (p = 0.031) and the mesioangular Winter’s position (p = 0.022) were associated with radiolucency in the distal aspect of the lower 3M, in the univariate logistic regression. Multivariable regression analysis revealed just one factor significantly associated with radiolucency: age (OR 1.05; CI 1.01–1.11; p = 0.036) (Table 2).

**Table 2:** Frequency distribution plus univariate/multivariate logistic regression model: radiolucency in the 3M distal and independent variables.

	Total (n)	Radiolucency in the distal aspect of the 3M (mm)		Univariate OR (CI 95%)	*p*-value	Multivariate OR (CI 95%)	*p*-value
		0 to 1.99	2.00 to 4.10				
**Sex**							
Male	102	91	11	1		–	–
Female	186	160	26	1.34 (0.64–2.95)	0.439	–	–
Age (years)	288	23.28 (± 5.75)	21.89 (± 3.70)	1.05 (1.01–1.11)	0.031**	1.05 (1.01–1.11)	0.036**
Angulation	288	22.25 (± 31.16)	7.38 (± 17.59)	1.00 (0.99–1.01)	0.689	–	–
Distance (mm)	288	3.54 (± 2.43)	2.50 (± 1.56)	0.99 (0.85–1.14)	0.970	–	–
**Third molar**							
38 (left)	138	117	21	1		–	–
48 (right)	150	134	16	0.66 (0.32–1.32)	0.251	–	–
**Pell and Gregory (occlusal surface)**							
I	48	42	6	1		–	–
II	240	209	31	0.83 (0.41–1.68)	0.604	–	–
III	0					–	–
**Pell and Gregory (ramus)**							
A	121	104	17	1		–	–
B	167	147	20	1.03 (0.43–2.89)	0.937	–	–
C	0	0	0			–	
**Winter classification**							
Vertical	129	105	24	1		1	
Mesioangular	93	86	7	0.35 (0.13–0.82)	0.022**	0.45 (0.17–1.02)	0.074
Horizontal	34	28	6	3.78e−08 (1.23e−170–9.72e+17)	0.988	–	–
Distoangular	69	32	37	0.82 (0.28–2.07)	0.691	–	–

Data are presented as n of subjects (%) or as mean ± SD.

*OR* odds ratio, *CI 95%* 95% of confidence interval.

*p < 0.20; **p < 0.05.

## Discussion

The present study revealed that among patients with partially erupted lower 3Ms, advanced ages, and greater angulations between the 3M and 2M were independent factors that contributed to the presence of a blurry bone aspect, horizontal bone loss, or vertical bone loss in the alveolar bone crest between 3 and 2M. In addition, older age was positively associated with radiolucency in the distal aspect of lower 3M values.

Although there is evidence that 2Ms adjacent to 3Ms may show periodontal problems, such as gingival inflammation^2,4^, periodontal pocket^3^, and alveolar bone resorption^3,5^, to the authors’ knowledge, this is the first paper to demonstrate an association between age and alveolar bone crest status. The radiographic examination assessing alveolar bone crest levels is a very useful adjunct diagnostic to the clinical periodontal examination^12^. Periodontal diseases have some radiographic signs that usually start as a blurry bone aspect (“fuzziness”) or a lamina dura discontinuity in the alveolar bone crest region^12^. Then, the destruction will extend to the interdental septa and end up in a height reduction of the bone crest^12^. In this sense, the bone crest alteration should be considered the initial process of periodontal disease.

According to the statistical results, after controlling for confounding factors, for each year increased in the individual’s age, the chance of alveolar bone crest alteration increases by 15%. Therefore, age should be considered an important predictor for bone crest alterations and, consequently, an important reason to consider prophylactic third molar removal^1^. On the other hand, sufficient space in the dental arch must be considered for decision-making on prophylactic 3M removal once this variable can predict the 3M full eruption^15^.

The decision of lower 3M prophylactic removal is still a topic of wide discussion. Dental surgeons often have difficulties in the decision-making: remove the 3M as a prophylactic approach or wait until the development of associated pathologies as a reason for the removal? Some studies elucidate reasons to indicate 3M removal, such as caries, periodontitis, and pain^1,2,5,16,17^. A current systematic review concluded that the evidence comparing the prophylactic removal of impacted lower 3M with its retention is very limited^18^. Although sufficient evidence is lacking, the authors suggest that prophylactic removal may be the most cost-effective strategy^18^. However, these results are related to impacted teeth and systematic reviews evaluating the effectiveness of removing partially erupted teeth were not found. Thus, the lack of reliable evidence hinders the decision-making process. In this sense, this study elucidates new findings based on periapical radiographs’ analysis of the alveolar bone crest in the 3M region. The findings of this study are an additional consideration for both surgeons and patients when deciding whether to prophylactically extract or retain 3Ms.

When performing radiologic investigation in an asymptomatic 3M region, at least three radiographic signs can determine hard tissue alterations and indicate the necessity of surgical removal of the tooth: marginal bone loss in the distal aspect of adjacent 2M; the increased radiolucent area surrounding the 3M crown; and bone resorption in the adjacent 2M^8,19^. Greater angulations between 3 and 2M promote greater distances between these teeth and lead to the occurrence of food impaction^20^. Some studies reported an increased local inflammation and bone resorption related to this impacted food^21,22^, which may be observed clinically^9^. The present study showed that for each increased degree in the 3M angulation, keeping constant the other variables, the chance of comprising the bone crest increases by 3%. Some studies have reported that most pathological changes presented in panoramic radiographs were associated with mesioangular and horizontally impacted 3Ms, considering Winter’s classification^8,19^, which corroborates the results of this study. Few studies in this field describe the 3M angulation as a continuous variable. However, the literature suggests the advisability of using an objective measurement method to minimize the error introduced by observer interpretation^23^.

Specifically, pathological changes in radiographs as pericoronal radiolucency of more than 2.0 mm in the distal aspect, is a generally accepted reason for the extraction of impacted lower third molars^24^. The present study showed that only the age variable was significantly associated with radiolucency between the distal aspect of the lower 3M crown and mandibular ramus in the final multivariable regression model. Thus, older patients presented wider radiolucency in the distal aspect of lower 3M crown when compared to younger ages. According to previous studies^25,26^, the earlier diagnosed the necessity to remove a 3M that causes periodontal alterations distal to adjacent 2M, the greater will be the chance of reducing the local inflammatory activity. A study showed that this inflammatory activity may decrease from 77 before the surgery to 23% after 3M removal in young patients^25^. Thus, earlier radiographic examinations as a complement to clinical exam in periodontal conditions will help guide clinical assertive recommendations, especially considering asymptomatic teeth.

Cone-beam computed tomography is the gold standard radiographic method to analyze third molars and associated structures^19^, however, it has a high cost. The two most used methods to evaluate the regional bony anatomy when planning a surgery is the panoramic and periapical radiographs^12^. However, a study compared the measurements on panoramic and periapical radiographic methods with the real measurements of the 3M region and, although no statistically significant difference was found, distortions on panoramic exams were slightly greater than on periapical^27,28^. Another study suggested a full-mouth survey of periapical radiographs as the gold-standard for periodontal diagnosis^25^. Considering the radiation exposure, a single panoramic radiograph may sufficiently represent the periodontal status of an individual. However, as the interest of the present study was to evaluate only the lower 3M region, it can be suggested that the present evidence, which is based on periapical radiographs, is sufficient to indicate the presence or absence of periodontal pathologies with considerable accuracy without exceeding radiation exposure.

In addition, considering the ALARA principle (“as low as reasonably achievable”), where it should be avoided to expose a patient even to a small dose of radiation if receiving that dose has no benefits^29^, it is not justifiable to use a panoramic radiography to analyze only one lower 3M. Some undesired events may occasionally occur, such as with those patients that cannot tolerate the periapical X-ray film and thus it may be necessary to retake the exam. In these cases, the three main protective measures of the ALARA principle (time, distance, and shielding) should be followed precisely to avoid unnecessary X-ray exposure^29^. Further, X-ray positioners are also a great alternative to avoid mistakes when taking a periapical radiography.

Although periapical radiograph is not the best imaging exam to verify pathological conditions related to the 3M region^20^, it is a largely used method in clinical practice due to its low cost, easiness, and necessary low amount of equipment^27,28^. In addition, in the institution where this study was conducted, periapical radiographs are the required preoperative exam because they can be executed in the university with no costs to the patients. Periapical radiographs were the images available to conduct this study and investigate the target conditions. In future studies, the periodontal diagnosis must consider the association between radiographic and clinical findings.

Some limitations of the present study should be highlighted. The prevalence rate of periodontal pockets in the distal aspect of 2Ms adjacent to 3Ms used to calculate the sample size, considered patients from the United States, and it may be not representative of the population included in this study. Different techniques and executors may be adopted to perform the radiographic exams resulting in possible differences between time of X-ray exposure and incidence direction. Moreover, to precisely assess possible anatomic alterations caused by 3Ms, it would be necessary a random sample of a generalized population, but there is a limitation to perform X-ray exams without consistent indication, for ethical reasons. Thus, for patients with partially erupted 3Ms, it is recommended radiographic and clinical monitoring to verify the necessity of prophylactic or resolutive removal of these teeth. Finally, this is a cross-sectional study based only on periapical radiographs. To further elucidate other conditions associated with the risk of wider radiolucency and bone crest involvement, longitudinal studies must be conducted. No temporal relationships among these variables can be determined regarding our findings. Furthermore, additional studies are required to develop predictive equations to estimate the periodontal performance when the 3M is present.

In conclusion, older patients, and greater angulations between the 3M and the adjacent 2M are more likely to develop alveolar bone crest alterations in this region. Also, age is associated with wider radiolucency in the distal aspect of lower 3Ms crown. It is suggested that these characteristics should be considered when evaluating a 3M for extraction because of future implications. The proposed null hypothesis was rejected, and association between the presence of a partially erupted lower 3M and alterations in the local bone structures was confirmed by this study.

## Data Availability

The datasets generated and/or analyzed during the current study are not publicly available because data were extracted from radiographs of records from patients, but are available from the corresponding author on reasonable request.

## Abbreviations

3 M: Third molar
2 M: Second molar
STROBE: Strengthening the Reporting of Observational Studies in Epidemiology
CEJ: Cementoenamel junction
